# Omega-6 Fatty Acids: A Sustainable Alternative to Improve Beef Production Efficiency

**DOI:** 10.3390/ani11061764

**Published:** 2021-06-12

**Authors:** Bruno Ieda Cappellozza, Reinaldo Fernandes Cooke, Kelsey Margaret Harvey

**Affiliations:** 1 Nutricorp, Araras 13601-000, SP, Brazil; cappellozza@nutricorp.com.br; 2 Department of Animal Science, Texas A&M University, College Station, TX 77843, USA; 3 Prairie Research Unit, Mississippi State University, Prairie, MS 39756, USA; kelsey.harvey@msstate.edu

**Keywords:** beef cattle, growth, health, reproduction, omega-6 fatty acids

## Abstract

**Simple Summary:**

The global beef industry is currently challenged with improving production efficiency while fostering judicious use of limited natural resources. Sustainable management systems are warranted to ensure that worldwide demands for beef and ecological stewardship are met. Supplementing cattle with omega-6 fatty acids is a nutritional intervention shown to sustainably enhance productivity across different sectors of the beef industry. The purpose of this review is to discuss recent research that describes the advantages of supplementing omega-6 fatty acids on traits that are critical to beef production efficiency, including reproduction, immunocompetence, growth, and quality of carcass and beef products.

**Abstract:**

Global beef production must increase in the next decades to meet the demands of a growing population, while promoting sustainable use of limited natural resources. Supplementing beef cattle with omega-6 fatty acids (FAs) is a nutritional approach shown to enhance production efficiency, with research conducted across different environments and sectors of the beef industry. Omega-6 FA from natural feed ingredients such as soybean oil are highly susceptible to ruminal biohydrogenation. Hence, our and other research groups have used soybean oil in the form of Ca soaps (CSSO) to lessen ruminal biohydrogenation, and maximize delivery of omega-6 FA to the duodenum for absorption. In cow–calf systems, omega-6 FA supplementation to beef cows improved pregnancy success by promoting the establishment of early pregnancy. Cows receiving omega-6 FA during late gestation gave birth to calves that were healthier and more efficient in the feedlot, suggesting the potential role of omega-6 FA on developmental programming. Supplementing omega-6 FA to young cattle also elicited programming effects toward improved adipogenesis and carcass quality, and improved calf immunocompetence upon a stress stimulus. Cattle supplemented with omega-6 FA during growing or finishing periods also experienced improved performance and carcass quality. All these research results were generated using cattle of different genetic composition (*Bos taurus* and *B. indicus* influenced), and in different environments (tropical, subtropical, and temperate region). Hence, supplementing omega-6 FA via CSSO is a sustainable approach to enhance the production efficiency of beef industries across different areas of the world.

## 1. Introduction

The United Nations estimates that beef production will need to increase by 120% in the next decades to feed a growing world population, [1]. The resources for beef production will become even more limited as the planet population increases and urban areas expand. Hence, management systems that promote sustainable beef production are warranted to meet production demands while fostering ecological stewardship and judicious use of limited natural resources.

Beef cattle operations across the world typically rely on forage as the primary nutrient source, which represents nearly 81% of the feed supplied to cattle during their productive lives [2]. The seasonal nature of forage production leads to variation in quantity and quality of forage, requiring supplementation strategies designed to correct nutrient deficiencies [3]. Fat supplementation has been extensively investigated in beef production systems, particularly as a means to provide energy to cattle [4,5]. However, supplemental fats can have nutraceutical benefits to cattle beyond their energy contribution [6,7,8,9], particularly omega-6 fatty acids (FA) such as linoleic acid [5,7]. Research from our and other groups supplemented cattle with omega-6 FA using soybean oil as a source of linoleic acid in the form of Ca soaps to minimize ruminal biohydrogenation, and maximize delivery of omega-6 FA to the duodenum. Divalent cations such as Ca react with FA to form insoluble soaps that cannot be dissociated nor modified by the ruminal microbes. In turn, Ca soaps of FA are dissociated when exposed to the low pH of the abomasum, releasing the FA for duodenal absorption [10]. Therefore, the purpose of this review is to compile recent research on omega-6 FA supplementation via CSSO to beef cattle, and its potential to serve as a sustainable alternative to improve beef production efficiency. 

## 2. Supplemental Omega-6 FA and Female Reproduction

Cow–calf systems are the foundation for global beef industries by determining the number of cattle available for harvest. Reproductive failure is a key factor limiting productivity in cow–calf operations, and pregnancy loss has been recognized as one of the main reproductive challenges in cattle [11]. Although ≥90% of fertile beef females effectively conceive after a single service, nearly 50% remain pregnant 30 days after service and even less females give birth to a live calf [12]. Management interventions to minimize pregnancy loss and promote embryonic survival are thus warranted, including supplementation with omega-6 FA. Linoleic acid and its omega-6 derivatives, however, serve as a precursor for prostaglandin (PG) F2α synthesis [13], which triggers luteolysis and has embryotoxic effects during early gestation [14]. For this reason, omega-6 FA supplementation was initially perceived as detrimental to the reproductive performance of beef cows [5]. 

**Figure 1:**
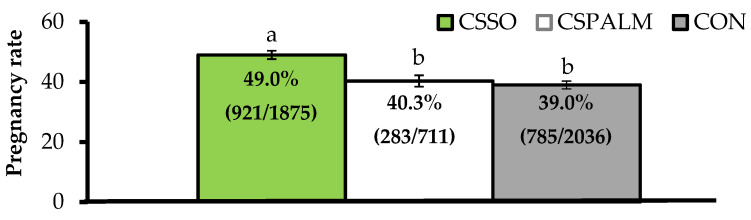
Pregnancy rates (as %) after artificial insemination in beef cows receiving Ca soaps of soybean oil (CSSO), Ca soaps of palm oil (CSPALM), or CON (unsupplemented) for 28 days after AI [15,16]. All values reported are least square means ± standard error (represented as error bars). Means with different superscripts differ (*p* ≤ 0.05). Information within parentheses indicate number of pregnant cows/total cows inseminated.

Differing from this latter concept, our research group reported that supplementing omega-6 FA via CSSO to beef cows after artificial insemination (AI) increased pregnancy rates by 25% [15,16]. Across a series of trials, grazing *Bos indicus* beef cows supplemented with 100 g/day of CSSO for 28 days beginning after AI had greater pregnancy rates compared with cows supplemented with 100 g/day of Ca soaps of palm oil (iso-caloric and iso-lipidic control rich in palmitic acid) or unsupplemented (CON) cows (Figure 1). These results provide evidence that omega-6 FA supplementation improved, and did not impair [5], the reproductive performance of beef females. Moreover, increased pregnancy rates resulting from omega-6 FA were associated with pregnancy establishment because CSSO was offered during the early embryonic period [17], and independent of their contribution to energy intake as CSPALM resulted in similar pregnancy rates to CON.

To provide biological support of the findings from Lopes et al. [15,16], Cooke et al. [18] investigated FA incorporation into reproductive tissues and physiological responses associated with pregnancy establishment. Grazing *B. indicus* beef cows were supplemented or not (CON) with 100 g/day of CSSO and slaughtered 19 days after AI. Cows receiving CSSO had greater incorporation of linoleic acid and its omega-6 derivatives into plasma, endometrium, corpus luteum, and conceptus. More specifically, CSSO supplementation increased intake and intestinal absorption of linoleic acid, which in turn was incorporated, elongated, desaturated, and accumulated into reproductive tissues, including as arachidonic acid in the conceptus (Table 1). These authors also evaluated factors associated with embryonic development and early pregnancy establishment on day 19 of gestation. These included conceptus size, mRNA expression of genes associated with pregnancy development in endometrial and luteal samples, and mRNA expression of interferon-tau (IFN-τ) by the conceptus; the conceptus-derived signal for maternal recognitions of pregnancy [17]. The increase in omega-6 FA accumulation, however, did not impact any of these variables, despite a tendency for increased IFN-τ concentration in uterine flushes collected from CSSO cows (10.9 vs. 7.3 ng/mL). Cows were slaughtered 19 days after AI to recover elongated conceptuses that still expressed IFN-τ mRNA [19] and provided enough tissue for both FA and mRNA expression analyses. The physiological processes responsible for pregnancy signaling to maternal tissues occur near days 15 to 17 of gestation [17]. Hence, Cooke et al. [18] evaluated maternal tissues and conceptuses after the critical period for pregnancy recognition, which prevented proper assessment of how omega-6 FA impacted expression of genes that mediate pregnancy establishment.

**Figure 2:**
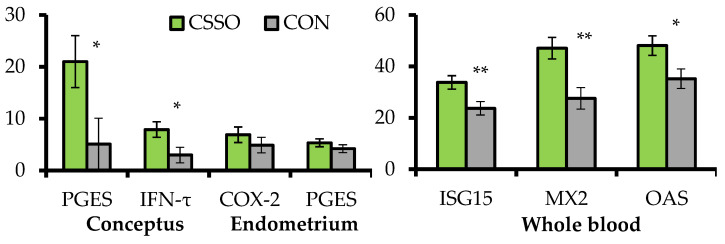
Expression of mRNA (relative fold change) in genes associated with pregnancy establishment in conceptus and endometrial samples collected on day 15 of gestation, and whole blood collected on day 20 of gestation from *B. indicus* cows receiving or not (CON; n = 10) Ca soaps of soybean oil (CSSO; n = 10) after artificial insemination. PGES = *prostaglandin E synthase*; *IFN-τ = interferon-tau; COX-2 = cyclooxygenase-2*; *ISG15 = interferon-stimulated gene 15*; *MX2 = myxovirus resistance 2*; *OAS* = *20*,*50*-*oligoadenylate synthetase*. All values reported are least square means ± standard error (represented as error bars). Within variable, ** *p* < 0.01 and * *p* ≤ 0.05. Adapted from Cipriano et al. [20].

To complement the results from Cooke et al. [18], Cipriano et al. [20] focused on conceptus- and endometrial-derived responses that mediate pregnancy signaling to maternal tissues on day 15 of gestation. Grazing *B. indicus* cows were supplemented or not (CON) with 100 g/day of CSSO beginning after AI. A subset of these cows were assigned to conceptus collection via transcervical flushing with saline followed by endometrial biopsy in the uterine horn ipsilateral to the corpus luteum 15 days after AI. The remaining cows were sampled for whole blood RNA extraction 20 days after AI, and pregnancy status was verified 28 days after AI. Supplementing omega-6 FA via CSSO increased conceptus length (2.58 vs. 1.15 cm) and mRNA expression of prostaglandin E synthase and IFN-τ by the conceptus, as well as mRNA expression of interferon-stimulated genes (ISG) in the whole blood (Figure 2). These results suggest that omega-6 FA supplementation enhanced conceptus development and IFN-τ synthesis during the pregnancy recognition period [17], corroborating the increased pregnancy rates to AI when CSSO was supplemented during early gestation [15,16]. The mRNA expression of ISGs have been used to gauge IFN-τ production and conceptus development from days 15 to 22 of gestation [21], given that IFN-τ synthesis upregulates mRNA expression of ISGs in circulating blood leukocytes [22]. Increased conceptus length and IFN-τ mRNA expression from supplemental omega-6 FA was associated with accumulation of arachidonic acid [18] and upregulation of prostaglandin E synthase mRNA in the conceptus. This enzyme converts PGH_2_ to PGE_2_ [23], which coordinates with IFN-τ endometrial functions that are critical for conceptus development and pregnancy signaling to maternal tissues [24]. In turn, CSSO supplementation did not impact the endometrial mRNA expression of *prostaglandin E synthase* and *cyclooxygenase-2* (Figure 2), suggesting that the effects of omega-6 FA on PG-related responses on day 15 of gestation may be specific to the conceptus due to heightened accumulation of arachidonic acid in this tissue and not in the endometrium [18].

Our initial efforts in characterizing the benefits of omega-6 FA to cattle reproduction were conducted with *B. indicus* cows reared in tropical conditions [15,16,18,20]. Pregnancy establishment and overall reproductive physiology differ between *B. indicus* and *B. taurus* females [25], and FA composition differs between tropical and temperate feed ingredients. Hence, Brandão et al. [26] conducted two trials evaluating omega-6 FA supplementation via CSSO to *B. taurus* cows in temperate conditions. In the first trial, grazing Angus cows were supplemented with 100 g/day of CSSO or prilled saturated fat (iso-caloric and iso-lipidic control; CON+) for 21 days after AI. Similar to the findings from Lopes et al. [15,16], pregnancy rates following AI were increased by 17% in cows supplemented with omega-6 FA (Table 2). The companion trial focused on conceptus- and endometrial-derived responses that mediate pregnancy signaling to maternal tissues with a design similar to Cipriano et al. [20], using Angus × Hereford cows that received 100 g/day of CSSO or CON+ beginning after AI. Supplementing omega-6 FA upregulated mRNA expression of IFN-τ by the conceptus and ISG in the whole blood, but did not increase conceptus length (11.3 vs. 11.4 cm for CSSO and CON, respectively) and mRNA expression of *prostaglandin E synthase*. Conceptus length across treatments was 11.4 ± 1.9 cm in Brandão et al. [26] and 2.4 ± 0.5 cm in Cipriano et al. [20], suggesting that *B. taurus* conceptus may be at an advanced stage of elongation on day 15 of gestation compared with *B. indicus* conceptus, and past the stage in which omega-6 FA impacts conceptus growth and expression of *prostaglandin E synthase*. Nevertheless, results from Brandão et al. [26] confirmed that omega-6 FA supplementation via CSSO to *B. taurus* cows also upregulated IFN-τ synthesis by the conceptus during the pregnancy recognition period, leading to increased pregnancy rates following fixed-time AI.

Collectively, supplementing omega-6 FA via CSSO increased incorporation of these FA into maternal and embryonic tissues and promoted IFN-τ synthesis by the conceptus during the maternal pregnancy recognition period, leading to increased pregnancy success in beef cows. These outcomes were generated across several research trials using nearly 6000 beef cows from different subspecies and managed in different environments, and were independent of the energy contribution of omega-6 FA given that iso-caloric and iso-lipidic control supplements were included. Hence, omega-6 FA supplementation is a nutritional alternative to enhance the reproductive efficiency of *B. taurus* and *B. indicus* beef cows reared in temperate and tropical environments.

## 3. Supplemental Omega-6 FA and Developmental Programming

The embryonic, fetal, and neonatal periods are the stages of life in which most developmental processes occur [27]. Nutrient supply during these periods exerts long-term consequences on the growth, development, and metabolic functioning of the offspring [28], leading to the concept of developmental programming [29]. Fetal developmental is sensitive to maternal nutrient status from oocyte maturation to parturition [30,31], and developmental plasticity remains susceptible to environmental stimuli during early postnatal life [32]. Dietary FAs provide a specific opportunity to nutritionally modulate developmental programming, as they differentially regulate expression of genes across metabolic tissues. For example, omega-3 FA limits adipose tissue accumulation by suppressing adipocyte differentiation [33,34], whereas omega-6 FA has been described as proadipogenic [35,36]. The fetal stage is critical for skeletal muscle and intramuscular adipocyte development [37,38]; hence, omega-6 FA supplementation during gestation can potentially enhance adipogenesis and thereby sites for marbling formation later in life [39].

### 3.1. Supplementing Omega-6 FA to Beef Cows during Gestation 

The majority of developmental research conducted to date has focused on energy and protein intake, although specific nutrients such as dietary FA are critical for optimal fetal and early-life development [40]. Our research group was the first to investigate the impacts of supplementing dietary FA to gestating beef cows on the post-natal performance of their offspring [41]. This initial project used beef cows supplemented with omega-3 and omega-6 FA (190 g/cow daily mixture of Ca soaps of polyunsaturated FA) or with a iso-caloric and iso-lipidic control treatment during the last trimester of gestation [41]. Calves born to cows supplemented with omega-3 and omega-6 FA had greater average daily gain (ADG) in the feedlot, and increased hot carcass weight (HCW), marbling score, and *longissimus* muscle (LM) area compared with cohorts from control cows. These results are indicative of programming effects on postnatal growth resulting from omega-3 and omega-6 FA [31], although the specific mechanisms underlying these outcomes warrant further investigation. As previously mentioned, omega-3 and omega-6 FA appear to have opposing effects on adipogenesis [33,34,35,36], and both sources of FA were fed to cows by Marques et al. [41]. Hence, Brandão et al. [42] focused on omega-6 FA supplementation to gestating beef cows based on their proadipogenic activities [35,36]. These authors supplemented beef cows with 200 g/day of CSSO or prilled saturated fat (CON+) throughout their last trimester of gestation. As expected, cows receiving CSSO had a greater plasma concentration of linoleic acid and total omega-6 FA compared with CON+ at calving (Table 3). Similar results were noted in the plasma FA profile of calves at birth (Table 3), given that maternal circulating FAs are transferred to the fetus via the placenta [42,43]. The concentration of immunoglobulin G in the colostrum and in plasma of calves were also increased by omega-6 FA supplementation (Table 3), which has life-long consequences on offspring immunocompetence and development [44]. The immunomodulatory properties of omega-6 are also expected to impact fetal immune system development [24]. Accordingly, incidence of bovine respiratory disease (BRD) and the number of antimicrobial treatments to recover from this disease were less in calves from CSSO cows compared with CON+ cohorts (Table 4), denoting the benefits of supplemental omega-6 FA during gestation on offspring life-long immunocompetence. 

Brandão et al. [42] also examined the mRNA expression of genes associated with adipogenesis and muscle development in the LM of newborn calves, and reported that calves born from CSSO supplemented cows had greater LM mRNA expression of *adipocyte fatty acid-binding protein 4* (FABP4), *stearoyl-CoA desaturase* (SCD), and *peroxisome proliferator-activated receptor gamma* (PPAR-γ) compared with CON+ calves (Table 4). Expression of PPAR-γ is stimulated by omega-6 FA [45,46], which regulates adipogenesis in bovine intramuscular adipose tissue [47] through induction of additional adipogenic genes including FABP4 and SCD [48,49]. Therefore, Brandão et al. [42] concluded that CSSO supplementation to beef cows increased the supply of omega-6 FA to the fetus, resulting in increased expression of genes involved in adipocyte differentiation and lipogenesis. These outcomes, however, were not translated into increased carcass marbling upon slaughter (Table 4). Expression of *myogenic differentiation 1* and *myogenin* mRNA in the LM were also upregulated at birth in calves from CSSO cows (Table 4). These are myogenic regulatory factors expressed by myocytes, and influence postnatal muscle growth through differentiation and fusion with existing muscle fibers [50,51]. Brandão et al. [42] postulated that supplementing omega-6 FA to gestating cows promoted the differentiation and development of fetal muscle cells via proinflammatory pathways [52], leading to enhanced myoblast proliferation and thereby myogenesis [53,54,55]. Accordingly, LM area was increased upon slaughter in calves from CSSO cows compared with CON+ cohorts (Table 4), suggesting that hastened development of fetal muscle fibers from omega-6 FA supplementation persisted postnatally when offspring were offered anabolic feedlot diets [56]. Together, results from Marques et al. [41] and Brandão et al. [42] provide initial evidence that omega-6 FA supplementation to gestating cows can be used to sustainably improve beef production efficiency. Research is still warranted to further investigate how maternal FA supplementation impacts fetal adipocyte development and myogenesis, including the specific roles of omega-6 and omega-3 FA.

### 3.2. Supplementing Omega-6 FA to Beef Calves in Early Postnatal Life 

Organ development and tissue differentiation are not complete at birth in most mammals, and the potential for an organism to diverge from a developmental trajectory extends into early postnatal life [57]. The biological responses to a nutritional intervention applied in early postnatal life are defined as metabolic imprinting, and permanently alter physiological outcomes later in life [58]. Given the potential of omega-6 FA to stimulate adipocyte development and myogenesis during periods of developmental plasticity [42], research has also focused on supplementing omega-6 FA to cattle via CSSO during early-life to enhance carcass characteristics. Mangrum et al. [59] and Tipton et al. [60] reported enhanced early adipocyte hyperplasia and differentiation in cattle receiving supplemental omega-6 FA, denoted by decreased average intramuscular adipocyte diameter and increased hyperplasia of intramuscular adipocytes. However, Mangrum et al. [59] reported increased lipid content in striploin steaks and improved marbling upon slaughter in cattle supplemented with omega-6 FA at 5 months of age, whereas the same outcomes were not observed with omega-6 FA supplementation started at 7 months of age [60]. Therefore, younger animals appear to be more susceptible to the metabolic imprinting effects from omega-6 FA, and supplementing these FAs earlier in life may yield a greater number of undifferentiated stem cells driven toward adipogenic confirmation and differentiation [60,61].

Schubach et al. [62] investigated in a 2 × 2 factorial design the impacts of omega-6 FA supplementation via CSSO to beef steers via creep feeding (CF; 2 months of age), and/or during a 40-day post-weaning period (7 months of age) on carcass development and quality. Steers receiving omega-6 FA via CF had greater mRNA expression of FABP4, *fatty acid synthase*, PPAR-γ, and SCD in LM samples collected in the feedlot (Table 5), when cattle were exposed to high-energy anabolic diets and lipogenesis was substantial [56], compared to cohorts receiving prilled saturated fat (CON+) during CF. Supplementing omega-6 FA post weaning had no impact on muscle gene expression in the feedlot, and providing omega-6 FA during both CF and post weaning did not yield additive benefits [62]. Hence, supplementation of omega-6 FA during a period of elevated epigenetic and developmental plasticity elicited alterations in mRNA expression of LM genes associated with adipogenic activities later in life, which are suggestive of a metabolic imprinting effect [39,61]. These alterations, however, did not translate into improved performance or carcass traits upon slaughter (Table 5). Therefore, omega-6 FA supplementation via CSSO during early-life appears to promote intramuscular adipogenic activities that persist later in life, although its benefits to carcass marbling and quality were inconsistent and require further investigation. 

## 4. Supplemental Omega-6 FA to Growing and Finishing Cattle

Weaning and feedlot receiving are two of the most stressful events in the beef production cycle, when cattle are exposed to a variety of physiological and physical stressors, including road transport, exposure to novel diets and environments, and comingling with new animals [63]. The combination of all of the stressors stimulates neuroendocrine and inflammatory reactions that directly impair cattle immunocompetence and productivity, leading to BRD incidence and reduced performance upon feedlot arrival [64]. Hence, strategies to increase the immunocompetence of cattle during the initial phases of the feedlot are warranted, including the use of omega-6 FA based on its immunomodulatory properties [65]. Research from our group demonstrated that omega-6 FA supplementation via CSSO to cattle upon feedlot arrival decreased plasma concentrations of inflammatory markers, but reduced feed intake and subsequent cattle ADG [8]. For this reason, our group evaluated omega-6 FA supplementation prior to feedlot arrival, by supplementing CSSO during a post-weaning preconditioning program [9]. Steers supplemented with omega-6 FA via CSSO during preconditioning had a greater feedlot-received ADG, which was attributed to reduced plasma concentrations of proinflammatory cytokines (Table 6). Moreover, CSSO steers had improved carcass marbling upon slaughter, which was associated with greater ADG upon feedlot arrival and potentially with metabolic imprinting effects, as omega-6 FA was supplemented when steers were 6 months old [9]. Hence, omega-6 FA supplementation prior to feedlot arrival should also be considered as a nutritional intervention to improve initial health and performance of feedlot cattle. 

Beef cattle are typically backgrounded on pasture after weaning in areas where forage is available for grazing [66], although supplemental nutrients are often required in this practice to meet the requirements of growing cattle [67]. Hess et al. [5] reviewed multiple studies in which omega-6 FA was supplemented to grazing cattle, but using grains and oilseeds highly susceptible to ruminal biohydrogenation [10]. To fill this gap in knowledge, Cappellozza et al. [68] evaluated performance and nutrient intake of grazing *B. indicus* bulls supplemented with omega-6 FA via CSSO. In this study, ADG was increased in bulls offered a grain-based supplement at 0.3% of their body weight fortified with omega-6 FA compared with bulls receiving an iso-caloric and iso-nitrogenous control supplement (0.92 vs. 0.81 kg/day, respectively). These authors also noted that bulls supplemented with omega-6 FA consumed less water (4.11 vs. 4.96% of body weight), and hypothesized that this outcome was due to reduced ruminal caloric increment from inclusion of CSSO into the supplement [68]. More specifically, CSSO partially replaced corn to maintain the supplement’s iso-caloric and iso-nitrogenous status, whereas ruminal fermentation of starch resulted in greater heat production compared with rumen-inert fats [68,69]. 

Another area of limited research is the inclusion of omega-6 FA into feedlot diets, as these FA from natural sources can disrupt ruminal function, feed intake and efficiency, and overall cattle performance [5]. The use of CSSO may partially alleviate these concerns, as supplementing Ca soaps of cottonseed oil improved feed efficiency of feedlot *B. indicus* bulls compared with cohorts receiving isocaloric and isonitrogenous diets [70]. Accordingly, Nascimento et al. [70] investigated the inclusion of omega-6 FA via CSSO, or a mixture of palm, soybean, and cottonseed oils fed as Ca soaps into feedlot diets (CSMIX). Supplemented CSSO or CSMIX increased energy intake, feed efficiency, ADG, and carcass merit of *B. indicus* finishing bulls compared with cohorts not receiving supplemental fat (Table 7). In turn, cattle performance and carcass traits were not improved by omega-6 FA supplementation via CSSO compared with the saturated + monounsaturated FA provided by the CSMIX (Table 7). Therefore, omega-6 FA inclusion via CSSO to feedlot diets improved cattle performance and efficiency by increasing the energy density of the diet, whereas a combination of saturated + monounsaturated FA appears to be more favorable for feedlot productivity and carcass quality [71,72].

## 5. Conclusions

This review compiled recent research on omega-6 FA supplementation via CSSO to beef cattle, and its benefits to production efficiency across different environments and sectors of the beef industry. Supplementing omega-6 FA increased the reproductive efficiency of beef cows by promoting the processes associated with early pregnancy establishment. Omega-6 FA also elicited positive effects during periods of developmental plasticity, such as gestation and early postnatal life. Supplementing omega-6 FA to beef cows during late gestation resulted in alterations in tissue differentiation and improved health and productivity of offspring. Similar effects on developmental programming were noted when omega-6 FA was supplemented to young calves. Lastly, supplementing omega-6 FA to growing cattle receiving forage-based diets resulted in enhanced immunocompetence, growth, and carcass merit, although such benefits were not evident when omega-6 FA was provided to feedlot cattle consuming high-concentrate diets. Collectively, this review provides research-based evidence that omega-6 FA supplementation via CSSO is a sustainable approach to improve beef production efficiency.

## Figures and Tables

**Table 1:** Concentrations of fatty acids (FA) in samples collected on day 19 of gestation from *B. indicus* beef cows receiving or not (CON; n = 9) Ca soaps of soybean oil (CSSO; n = 9) after artificial insemination. Values reported are least square means ± standard error. Adapted from Cooke et al. [18].

Item	CON	CSSO	*p* =
Plasma (mg of FA/g of plasma)			
Linoleic acid	0.275 ± 0.022	0.540 ± 0.021	<0.01
Arachidonic	0.023 ± 0.005	0.025 ± 0.004	0.74
Total omega-6 FA	0.296 ± 0.023	0.565 ± 0.022	<0.01
Endometrium (mg of FA/g of tissue)			
Linoleic acid	0.144 ± 0.043	0.358 ± 0.044	<0.01
Arachidonic	0.241 ± 0.061	0.266 ± 0.061	0.77
Total omega-6 FA	0.549 ± 0.136	0.938 ± 0.136	0.05
Corpus luteum (mg of FA/g of tissue)			
Linoleic acid	3.935 ± 0.543	7.035 ± 0.543	<0.01
Arachidonic	4.731 ± 0.349	4.942 ± 0.350	0.67
Total omega-6 FA	12.72 ± 1.19	17.78 ± 1.19	<0.01
Conceptus (mg of FA/g of tissue)			
Linoleic acid	0.022 ± 0.059	0.174 ± 0.062	0.08
Arachidonic	0.086 ± 0.043	0.312 ± 0.043	<0.01
Total omega-6 FA	0.384 ± 0.753	2.045 ± 0.755	0.13

**Table 2:** Pregnancy rates and expression of mRNA (relative fold change) of genes associated with pregnancy establishment in *B. taurus* cows receiving Ca soaps of soybean oil (CSSO) or prilled saturated fat (CON+) after artificial insemination. Values reported are least square means ± standard error. Adapted from Brandão et al. [26] ^1^.

Item	CON+	CSSO	*p* =
Pregnancy rates to AI (n = 11/treatment), %	51.7 ± 4.1	60.2 ± 4.2	0.01
Physiological responses (n = 9/treatment)			
Endometrium, mRNA expression			
*Cyclooxygenase-2*	5.11 ± 1.32	4.88 ± 1.33	0.89
*Prostaglandin E synthase*	7.40 ± 1.05	5.76 ± 1.15	0.30
Conceptus, mRNA expression			
*Interferon-tau*	12.1 ± 3.6	21.3 ± 3.4	0.05
*Prostaglandin E synthase*	2.50 ± 0.49	2.22 ± 0.48	0.69
Whole blood, mRNA expression			
*Interferon-stimulated gene 15*	29.8 ± 4.9	43.1 ± 4.3	0.04
*Myxovirus resistance 2*	20.1 ± 2.8	20.2 ± 2.5	0.98
*20,50-oligoadenylate synthetase*	18.3 ± 2.9	26.8 ± 2.6	0.03

^1^ Conceptus and endometrial samples were collected on day 15 of gestation and whole blood was collected on day 20 of gestation, whereas pregnancy rates were diagnosed 28 days after AI.

**Table 3:** Concentrations of fatty acids (FA) and immunoglobulin G (IgG) in cows and their calves upon calving. Cows were supplemented with Ca soaps of soybean oil (CSSO; n = 52) or prilled saturated fat (CON+; n = 52) during the last trimester of gestation. Values reported are least square means ± standard error. Adapted from Brandão et al. [42].

Item	CON+	CSSO	*p* =
Cow plasma upon calving, μg/mL			
Linoleic (18:2 n-6)	145 ± 12	342 ± 12	<0.01
Arachidonic (20:4 n-6)	13.3 ± 0.7	19.2 ± 0.7	<0.01
Total omega-6	202 ± 13	408 ± 13	<0.01
Calf plasma at birth, μg/mL			
Linoleic (18:2 n-6)	24.5 ± 4.3	41.9 ± 4.2	<0.01
Arachidonic (20:4 n-6)	7.98 ± 0.70	11.6 ± 0.68	<0.01
Total omega-6	37.6 ± 4.5	59.5 ± 4.4	<0.01
Total FA	311 ± 15	319 ± 15	0.73
Colostrum IgG upon calving, mg/mL	373 ± 16	423 ± 15	0.02
Calf plasma IgG 24 h after birth, mg/mL	55.7 ± 9.0	97.9 ± 9.0	<0.01

**Table 4:** Expression of mRNA (relative fold change) of *longissimus* muscle (LM) genes at birth, incidence and treatments against bovine respiratory disease (BRD) in the feedlot, and carcass traits of calves born from cows that received diets supplemented with Ca soaps of soybean oil (CSSO; n = 52) or prilled saturated fat (CON+; n = 52) during the last trimester of gestation. Values reported are least square means ± standard error. Adapted from Brandão et al. [42].

Item	CON+	CSSO	*p*-Value
LM muscle at birth, mRNA expression			
*Adipocyte fatty acid-binding protein*	35.0 ± 14.1	73.9 ± 14.6	0.03
*Myogenic differentiation 1*	13.3 ± 3.0	22.6 ± 3.1	0.02
*Myogenin*	7.03 ± 1.0	9.77 ± 1.1	0.04
*Peroxisome proliferator-activated receptor-γ*	3.16 ± 0.54	4.55 ± 0.58	0.07
*Stearoyl-CoA desaturase*	3.43 ± 0.32	4.54 ± 0.36	0.05
Treated for BRD in the feedlot, %			
Once	40.5 ± 6.7	28.4 ± 6.7	0.16
Twice	19.2 ± 4.71	5.64 ± 4.68	0.03
Number of antimicrobial treatments required	1.49 ± 0.10	1.18 ± 0.11	0.05
Carcass traits upon slaughter			
LM, cm^2^	79.6 ± 1.0	82.4 ± 1.1	0.03
Yield grade	3.76 ± 0.07	3.68 ± 0.07	0.43
Marbling	526 ± 15	510 ± 15	0.47
Backfat, cm	2.46 ± 0.14	2.40 ± 0.14	0.76

**Table 5:** Expression of mRNA (relative fold change) of *longissimus* muscle (LM) genes in the feedlot, and carcass traits of steers supplemented with Ca soaps of soybean oil (CSSO; n = 16) or prilled saturated fat (CON+; n = 16) from 2 to 4 months of age. Values reported are least square means ± standard error. Adapted from Schubach et al. [62].

Item	CON+	CSSO	*p*-Value
LM muscle, mRNA expression			
*Adipocyte fatty acid-binding protein*	38.5 ± 8.4	72.5 ± 8.3	0.02
*Fatty acid synthase*	124 ± 18	210 ± 19	0.02
*Peroxisome proliferator-activated receptor-γ*	5.01 ± 0.74	8.20 ± 0.75	0.01
*Stearoyl-CoA desaturase*	96.5 ± 17.4	202 ± 17.7	<0.01
Carcass traits upon slaughter			
LM area, cm^2^	80.0 ± 1.3	79.3 ± 1.4	0.72
Yield grade	3.48 ± 0.09	3.55 ± 1.00	0.62
Marbling	493 ± 15	470 ± 16	0.32
Backfat, cm	1.50 ± 0.04	1.52 ± 0.05	0.69

**Table 6:** Performance and health responses from steers supplemented or not (CON; n = 6) with Ca soaps of soybean oil (CSSO; n = 6) for 28 days prior to feedlot arrival (day 0). Values reported are least square means ± standard error. Adapted from Cooke et al. [9].

Item	CON	CSSO	*p* =
Plasma tumor necrosis alpha, pg/mL (log)			
Day 0 (arrival)	1.74 ± 0.21	1.91 ± 0.21	0.58
Day 1	1.88 ± 0.23	2.00 ± 0.22	0.67
Day 3	2.23 ± 0.20	1.55 ± 0.20	0.03
Feedlot average daily gain, kg/d			
Initial phase (day 1 to 144)	1.17 ± 0.02	1.25 ± 0.02	0.02
Final phase (day 145 to slaughter)	2.10 ± 0.05	2.09 ± 0.05	0.86
Carcass traits			
Hot carcass weight, kg	394 ± 6	402 ± 6	0.31
*Longissiumus* muscle area, cm^2^	94.7 ± 1.5	92.0 ± 1.6	0.23
Yield grade	3.16 ± 0.10	3.48 ± 0.11	0.04
Marbling	444 ± 18	515 ± 19	0.01
Backfat, cm	1.55 ± 0.06	1.63 ± 0.06	0.38

**Table 7:** Performance and carcass traits of feedlot bulls supplemented or not (CON; n = 16) with Ca soaps of soybean oil (CSSO; n = 16) or a mixture of palm, soybean, and cottonseed oils (CSMIX; n = 15) until slaughter. Values reported are least square means ± standard error. Adapted from Nascimento et al. [70] ^1^.

Item	CON	CSSO	CSMIX	C1	C2
Performance					
Average daily gain, kg/d	1.14 ± 0.04	1.37 ± 0.05	1.48 ± 0.05	<0.01	0.11
Feed efficiency, g/kg	156 ± 3	168 ± 3	183 ± 3	<0.01	<0.01
Final body weight, kg	476 ± 6	508 ± 7	524 ± 7	<0.01	0.13
*Carcass traits*					
Hot carcass weight, kg	268 ± 4	284 ± 4	297 ± 4	<0.01	0.03
*Longissiumus* muscle area, cm^2^	67.8 ± 1.88	70.4 ± 1.94	75.4 ± 1.94	0.04	0.08
Backfat, cm	0.318 ± 0.035	0.439 ± 0.039	0.448 ± 0.040	0.01	0.87

^1^ C1 = CON vs. CSSO + CSMIX; C2 = CSSO vs. CSMIX.

## Data Availability

Not applicable.
